# Supplementary figures and images for: Voxel-wise deep learning segmentation of hydroxyapatite and iodine in spectral photon-counting CT: A quantitative phantom study
Source: PLoS One. 2026 Apr 9;21(4):e0346825. doi: 10.1371/journal.pone.0346825 (PMC13065060; doi:10.1371/journal.pone.0346825)

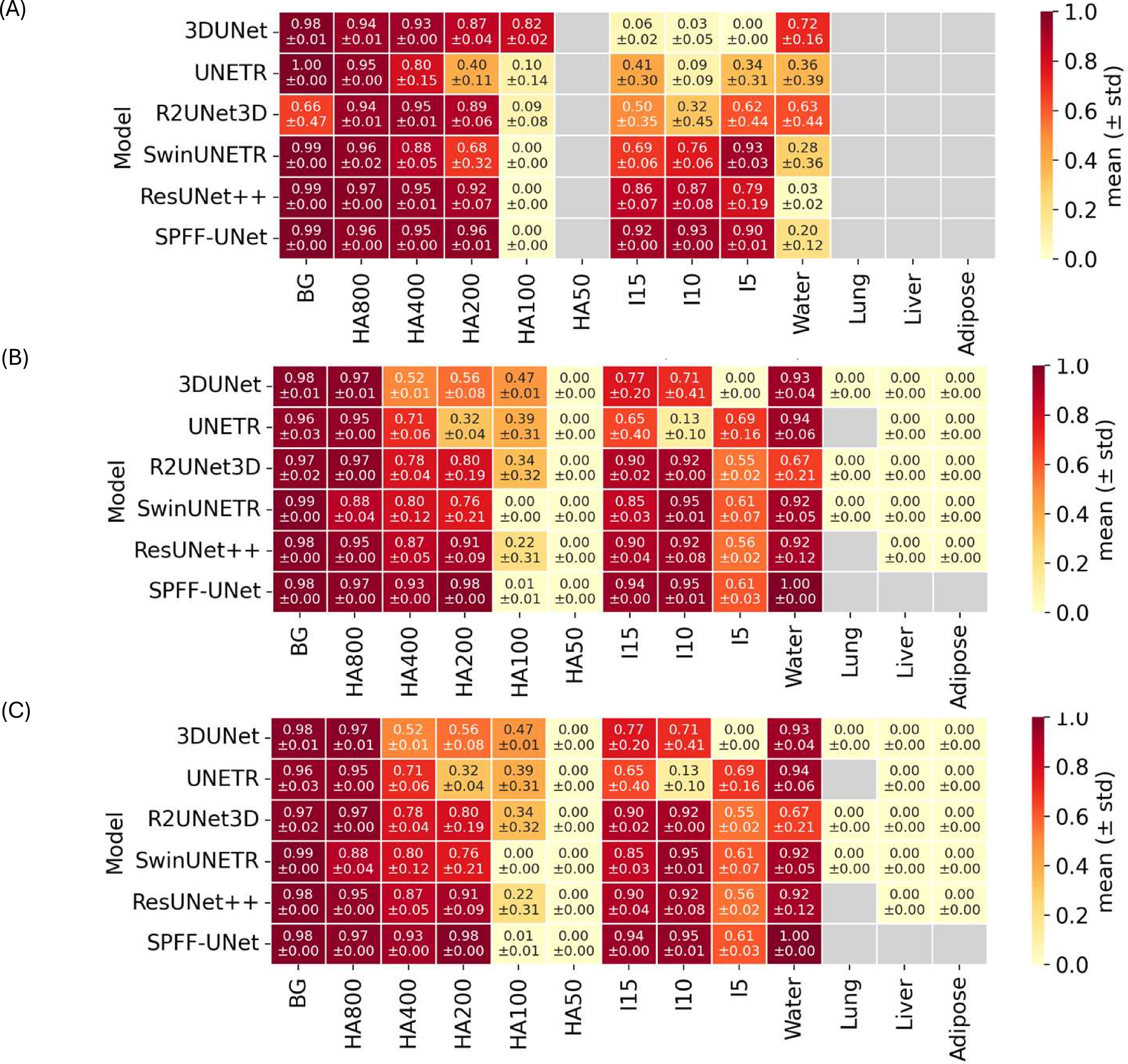

Supplement: S1 Fig — (A) Sensitivity. (B) Precision. (C) IoU. Rows are models; columns are classes. Values are mean ± SD across three seeds on the external test. Light–gray cells labeled N/A indicate classes absent from the ground truth and excluded from summaries; for these, Precision/IoU are undefined when no positives are predicted (TP = 0, FP = 0). For the absent soft–tissue classes, SPFF–UNet is consistently N/A, indicating it did not hallucinate labels. Several baselines instead show 0.00 ± 0.00 in the same columns—this occurs when TP = 0 but FP > 0, collapsing Precision and IoU to zero because false positives were produced for classes that should be empty. This behavior is consistent with our results.BG: Background. (TIF) [file pone.0346825.s001.tif]
