# Supplementary material for: Voxel-wise deep learning segmentation of hydroxyapatite and iodine in spectral photon-counting CT: A quantitative phantom study
Source: PLoS One. 2026 Apr 9;21(4):e0346825. doi: 10.1371/journal.pone.0346825 (PMC13065060; doi:10.1371/journal.pone.0346825)
Supplement: Attachment — Submitted filename: Response to Reviewers.pdf [file pone.0346825.s002.pdf]

Dear Editor and Reviewers,

We sincerely thank you for the careful evaluation of our manuscript and for the constructive comments and additional journal requirements. We greatly appreciate the time and effort invested in the review process. We have carefully considered all comments and revised the manuscript accordingly. Below we provide a point-by-point response to all items raised. All changes in the revised manuscript are highlighted in red for ease of reference.

Journal Requirements

1. PLOS ONE style requirements and file naming
   Response: We revised the submission files to conform to PLOS ONE style requirements and file-naming conventions, and prepared the revised manuscript using the PLOS ONE formatting templates provided by the journal.

2. Code sharing requirements
   Response: We confirm that all author-generated code underpinning the findings has been made publicly available without restrictions. The full code used for model training, evaluation, and figure generation has been archived on Zenodo (v1.0.0 DOI: 10.5281/zenodo.18797577; all versions DOI: 10.5281/zenodo.18797576) and is mirrored on GitHub (https://github.com/NF-91/spff-unet-spcct). This information is stated in the manuscript's Data Availability section.

3. Data availability ("available upon request" selection in submission form)
   Response: We updated the manuscript to ensure compliance with PLOS data policy. The dataset underlying the study is publicly available in IEEE DataPort (DOI: 10.21227/gbhn-nk95). Source data underlying the reported figures and tables are provided within the manuscript and its Supporting Information files. We updated the Data Availability statement accordingly.

4. Role of funders statement
   Response: We included the amended Role of Funder statement in the cover letter, as requested.

5. Copyright concerns for Figures 1–5 and S1
   Response: All figures have been reviewed and updated to contain only author-created content (no third-party icons/clipart/logos/screenshots). Some figures include example SPCCT phantom images from our study dataset, which was generated by the authors and is publicly available via IEEE DataPort (DOI: 10.21227/gbhn-nk95). All figures can be published under CC BY 4.0 and were generated directly from the data and outputs produced in this study.

6. Supplementary file type / legends for Supporting Information
   Response: We amended the file type of supplementary figures to "Supporting Information" in the submission system and ensured that each Supporting Information item has a corresponding legend listed in the manuscript after the references.

7. Reviewer-suggested citations
   Response: We reviewed relevant suggested/related works and added citations where appropriate to improve context and completeness.

8. Reference list completeness and correctness
   Response: We reviewed the reference list to ensure accuracy and completeness. To our knowledge, none of the cited references are retracted. Any additions/changes to references are reflected in the revised manuscript and are noted below where relevant.

Editor Comments

1. Please shorten the abstract. Try to make it more concise. It is not intelligibly presented. It exceeds the word limit.

   Response: Thank you for this helpful suggestion. We substantially shortened and rewrote the abstract to improve clarity, fluency, and concision while preserving the study objective, core methodology, principal findings, and main conclusion.

2. A concern by a reviewer is that if there are public datasets, how could the findings from this manuscript be generalized to public datasets? A short paragraph in the discussion section is recommended.

   Response: We agree that generalizability beyond the present phantom dataset should be discussed more explicitly. We added a paragraph to the Discussion clarifying that directly matched public benchmarks for five-bin SPCCT hydroxyapatite/iodine voxel-wise labeling are currently limited. We also note that related public spectral CT datasets exist (e.g., the 2022 AAPM deep-learning spectral CT grand challenge dataset~\cite{sidky2024aapm_dlspectralct_dataset,sidky2024aapm_report}), but these differ substantially in acquisition type, task definition, and target labels, and therefore do not provide a like-for-like external benchmark for the present study. We highlight this as an important direction for future work.

Reviewer #1

1. Data availability and reproducibility
   To fully align with PLOS ONE's data-sharing policy, the authors should include a clear Data Availability Statement specifying whether datasets, trained models, or code will be made publicly available, and where.

   Response: Thank you for this important suggestion. We added a clear Data Availability Statement to the manuscript. The dataset used in this study is publicly available in IEEE DataPort (DOI: 10.21227/gbhn-nk95). The full code used for model training, evaluation, and figure generation has been made publicly available and archived on Zenodo (v1.0.0 DOI: 10.5281/zenodo.18797577; all versions DOI: 10.5281/zenodo.18797576) and is mirrored on GitHub (https://github.com/NF-91/spff-unet-spcct). Source data underlying the reported tables and figures are provided within the manuscript and its Supporting Information files.

2. Clinical claims and framing
   While the discussion is generally cautious, a few statements could be further softened

to avoid any implication of near-term clinical applicability. Explicitly reiterating that no clinical claims are made and that in vivo validation is required would strengthen alignment with the journal's scope.

Response: We appreciate this suggestion and agree that the phantom-based nature of the study should remain central to the framing. We revised the Discussion and Conclusion to further soften statements that could be interpreted as implying near-term clinical applicability. We now explicitly reiterate that no clinical claims are made and that in vivo and multi-center validation will be required before any clinical translation can be considered.

3. Justification of classification vs. regression
   Although implicit in the motivation, a brief explicit discussion explaining why discrete concentration-aware classification was favored over continuous regression (commonly used in spectral CT) would improve clarity for a broad readership.

   Response: We thank the reviewer for this important point. We added a brief discussion clarifying why a classification formulation was used in this work rather than continuous regression. In brief, our goal was to generate directly interpretable voxel-wise concentration labels for predefined hydroxyapatite and iodine classes, whereas regression would require an additional post hoc discretization step for the specific decision context considered here.

4. Statistical generalizability
   The authors may wish to emphasize that the external test set consists of a single phantom scan and that reported variability reflects training stochasticity rather than population-level variance.

   Response: We agree and clarified this limitation explicitly. The revised manuscript states that the external test set comprises a single independent phantom scan and that the reported mean ± SD across seeds reflects variability due to repeated training (training stochasticity/model robustness), rather than population-level statistical uncertainty across independently sampled phantoms or patients.

Reviewer #2

Comment: The used dataset should be compared with globally used datasets to provide a stronger reference and a more robust scientific methodology, and should also be compared with another standard outside dataset to improve accuracy and confidence.

Response: We appreciate the reviewer's emphasis on external reference datasets and broader generalizability. We added a paragraph to the Discussion addressing this point. Specifically, we clarify that directly comparable public datasets for five-bin SPCCT voxel-wise hydroxyapatite/iodine concentration labeling are currently limited. We also discuss related public spectral CT resources (e.g., the 2022 AAPM deep-learning spectral CT grand challenge dataset~\cite{sidky2024aapm_dlspectralct_dataset,sidky2024aapm_report}) and explain why they are not directly task-matched to the present phantom study. This limitation is now

acknowledged more explicitly, and future validation on larger external and public datasets is highlighted as an important next step.
